# Characterization of VDR and CYP27B1 expression in the endometrium during the menstrual cycle before embryo transfer: implications for endometrial receptivity

**DOI:** 10.1186/s12958-020-00579-y

**Published:** 2020-03-17

**Authors:** Jing Guo, Shan Liu, Peng Wang, Haiying Ren, Yuan Li

**Affiliations:** Center for Reproductive Medicine, Beijing Chao-Yang Hospital, Capital Medical University, Workers’ stadium South Road 8, Chao-yang district, Beijing, China

**Keywords:** Vitamin D, Vitamin D receptor, CYP27B1, Endometrial receptivity, In-vitro fertilization

## Abstract

**Background:**

Molecular analyses of vitamin D in a typical cycling endometrium has received minimal research attention in the reproductive field. This study was designed to assess how expression of the endometrial vitamin D receptor (VDR) and CYP27B1, a vitamin D metabolizing enzyme, change during the menstrual cycle in women of reproductive age. In addition, this study explores the association between expression of vitamin D-VDR system and endometrial receptivity during the implantation window.

**Methods:**

Sixteen patients underwent standardized in vitro fertilization (IVF) treatment and freeze-all techniques. Before embryo transfer, total serum 25(OH) D levels were determined through blood samples and VDR, CYP27B1, HOXA10, and CYP19 expression were determined through endometrial samples. Endometrial receptivity was also assessed using an electron microscope.

**Results:**

We found that VDR protein expression was significantly lower throughout the endometrial secretory phase compared to the proliferative phase, while CYP27B1 expression remained constant during the menstrual cycle. During the implantation window, ultrastructural evaluation showed that higher serum vitamin D levels were associated with more mature pinopodes; VDR and HOXA10 protein expression were substantially elevated in pregnant women compared to non-pregnant women; and VDR protein levels were positively correlated with HOXA10 levels. In addition, serum vitamin D levels were positively correlated with VDR and HOXA10 protein levels in the endometrium.

**Conclusions:**

Women with increased VDR expression in the endometrium, especially during the implantation window of the menstrual cycle, were significantly more likely to be pregnant than women with decreased expression. Our results support the hypothesis that the Vitamin D-VDR system performs a role during the development of endometrial receptivity.

## Introduction

Vitamin D is well-known for, not only maintaining phosphorus and calcium homeostasis and promoting bone formation, but also for its role in cell proliferation, differentiation, and apoptosis; and the regulation of the immune system, hormones; and other biological processes [1]. In addition, vitamin D acts as a steroid hormone, in some cases, as it helps regulate the manifestation of numerous genes in reproductive tissues. Vitamin D is associated with modulation of the human reproductive process [2, 3].

Vitamin D obtained from diet and produced by sunlight via the skin are both metabolized by 25-hydroxylase, a liver enzyme, into 25-hydroxyvitamin D (25(OH)D). The enzyme CYP27B1 in the kidneys then hydroxylates 25(OH) D again and converts it into a bioactive form of the molecule, 1,25-dihydroxyvitamin D (1,25(OH)_2_D). Extrarenal CYP27B1 mRNA, as well as its protein expression, have been detected in the human endometrium, implying local vitamin D metabolism and influence on endometrial function [4].

1,25(OH)_2_D initiates biological responses by attaching to the vitamin D receptor (VDR), a transcription factor in the steroid hormone receptor superfamily. This active form mediates most of the physiological action of the vitamin. VDR binds with a retinoid X receptor to form a heterodimer structure. This complex then binds to vitamin D response elements (VDRE) in a precise gene promoter region to regulate the transcription of thousands of genes [5]. The physiological effects of 1,25(OH)_2_D are mediated through both genome-based and non-genome-based signaling pathways. The former refers to the formation of a complex with the vitamin D nuclear receptor in the cytoplasm, which is transported into the nucleus and acts as the ligand-dependent transcription factor to regulate target genes. The latter refers to binding that occurs at the membrane receptors located within caveolae and acts through a non-genomic signaling pathway [6].

As an endometrial receptivity marker, HOXA10 is an important molecule involved in embryo implantation. Altered expression of HOXA10 may lead to impaired endometrial receptivity and failure of the embryo to implant. In addition, 1,25(OH)_2_D increases HOXA10 mRNA and protein manifestation in human endometrial stromal cells. Therefore, the interaction between sex steroid hormones and vitamin D may regulate HOXA10, implicating the role of vitamin D during the peri-implantation period [7].

Endometrial or uterine expression of VDR suggests the functional role of the vitamin D-VDR system in uterine receptivity [6, 8–11]. Uterine hypoplasia and impaired folliculogenesis were observed in vitamin D receptor null mutant mice. In these mice, the aromatase (encoded by CYP19) activities and the gene expression of aromatase were both reduced in the ovary [12]. However, studies investigating the biological action of vitamin D in fertility and reproductive tissues have primarily used animal models. In addition, molecular analyses of the vitamin D-VDR system in a typical cycling endometrium and during pathophysiological conditions have received minimal research attention.

Although vitamin D insufficiency has been of interest to IVF researchers for many years, it remains unclear whether the endometrium or the oocyte is more susceptible to vitamin D deficiency. The in vitro fertilization and embryo-transfer (IVF-ET) population provides a valuable research model to analyze follicular development through embryo implantation and the role of vitamin D in different reproduction-related processes. Our previous study investigated associations between vitamin D blood concentrations and IVF-ET clinical outcomes for infertile women in southern China [13]. After controlling for multiple variables that might affect oocyte quality, our results indicated that serum 25(OH) D deficiency significantly impaired pregnancy chances in infertile women. This implies a role for vitamin D in endometrial receptivity and other pregnancy outcomes.

The endometrium undergoes proliferation, differentiation, and finally degeneration during a normal female menstrual cycle, which is primarily regulated by modifications to estrogen and progesterone concentrations [14]. CYP19, a key regulator of estrogen synthesis, is a rate-determining molecule that activates the reaction turning testosterone into estradiol [15]. Vitamin D, in its capacity as a steroid hormone with progesterone-like activity, may have a direct role in modifying the endometrium throughout the menstrual cycle [16, 17].

To date, very little research has been conducted regarding the molecular details of vitamin D activity in live human endometrial tissues. We hypothesize that the vitamin D-VDR system and associated molecules are expressed during the proliferation phase through the secretory phase within the human endometrial cycle. Additionally, we speculate that endometrial receptivity is associated with the expression of vitamin D-related molecules during the implantation window, which further affect pregnancy outcomes. In our current study, we established 1) the morphological changes in endometrial receptivity during the implantation window under different concentrations of vitamin D; 2) the expression and distribution of VDR, CYB27B1, HOXA10, and CYP19 proteins in the human endometrium throughout menstruation; and 3) the relationship between VDR, CYB27B1, HOXA10, and CYP19 expression in the endometrium and pregnancy outcomes.

## Materials and methods

### Study population

We recruited 16 women who underwent their first IVF-ET treatment cycle at the Reproductive Center of Beijing Chao-Yang Hospital of Capital Medical University. All patients were non-smokers, had regular menstrual cycles (28–30 days), and were residents of Beijing city. Patients were also ≤40 years old and had basal FSH concentrations ≤10 IU/L together with estradiol (E_2_) < 50 ng/ml. Causes of infertility included mild male factor and tubal infertility. Patients with endometriosis and adenomyosis were excluded. Patients with conditions known to affect implantation, like hydrosalpinx, endometrial polyp, uterine malformation, partial septate uterus, submucous myoma, and multiple uterine fibroids were identified using transvaginal sonography, and excluded.

### Treatment protocol

All patients underwent IVF treatment using the GnRH antagonist protocol for controlled ovarian stimulation (COS): an individualized daily dose of exogenous gonadotropins (Gonal-F, Merck) was issued for COS starting on day 3 of the menstrual cycle until human chorionic gonadotrophin (HCG) was administered. Evaluation of the ovarian response was performed utilizing transvaginal ultrasound, total serum E_2_, and luteinizing hormone (LH) assays. Cetrorelix acetate (Cetrotide; Merck) was provided when the follicular diameter ≥ 14 mm or LH level ≥ 4 IU/L. The dosage and duration of antagonist were determined according to the patient’s age, BMI, ovarian function, follicular development and hormone concentrations. Once at least 3 follicles grew to a length of 18 mm, ovulation was triggered using 0.2 mg triptorelin (Decapeptyl, Ferring) combined with 2000 IU HCG (recombinant, Ovitrelle, Merck).

Oocytes were aspirated 36 h later using vaginal ultrasonography. Typical lab procedures were utilized. Freeze-all techniques were utilized for all patients. If available, two cleavage embryos were transferred during the first frozen-thawed embryo transfer cycle. Protocols for endometrial preparation and luteal phase support described in our previous research were followed in this study [18]. Clinical pregnancy was diagnosed if elevated serum HCG levels were measured 2 weeks after embryo transfer and confirmed via transvaginal sonography.

### Endometrial biopsy and sample processing

An endometrial biopsy was performed during the menstrual cycle before embryo transfer. At the same time, transvaginal ultrasound confirmed that the bilateral ovaries recovered and the ascitic fluid completely absorbed after oocyte aspiration. The proliferative endometrial phase was defined as days 10–14, while the window of implantation (secretory endometrium phase) was defined as days 19–23 of the menstrual cycle. All endometrial samples were collected using a Pipelle sampler (Nuo De Medical) under sterile conditions. Portions of the endometrial biopsy products were immediately placed into 2.5% glutaraldehyde stationary liquid (Leagene) before undergoing electron microscopic analysis. Other endometrial products were immediately placed into a 10% neutral buffered formalin solution overnight and embedded into paraffin before undergoing immunohistochemistry. The remaining parts were stored using liquid nitrogen cryopreservation.

### Vitamin D determination and status

Serum 25(OH) D samples were obtained simultaneously during endometrial collection. Serum 25(OH) D samples were then centrifuged at 3000 g for 15 min and, the supernatant was stored at − 80 °C until assayed. We measured 25(OH) D levels via an enzyme-linked immunosorbent assay (AC-57F1, IDS, Germany). Intra-and inter-assay variation coefficients were 5.3 and 4.6%, respectively. We categorized 25(OH) D levels according to previously defined criteria from the Endocrine Society and Institute of Medicine (IOM). Individuals with serum 25(OH) D concentrations below 20 ng/ml were deemed vitamin D deficient, between 20 and 30 ng/ml were deemed insufficient, and above 30 ng/ml were deemed replete [19].

### Scanning electron microscope (SEM) and transmission electron microscope (TEM) analysis

3mm^2^ fresh tissue blocks were made and washed in phosphate buffer saline (PBS). Tissues were immediately put into a 2.5% glutaraldehyde stationary liquid at 20 °C for 2 h. Samples were then post-fixated in a 1% osmium tetroxide solution for 1 h at 20 °C and dehydrated in absolute ethyl alcohol. Samples were put into a critical point dryer (Quorum K850, UK), attached to the conductive carbon film double-sided adhesive, and placed on the ion sputtering apparatus for gold spraying. After that, samples were observed using a SEM (HITACHI SU8100, Japan).

Tissues previously fixed in a 2.5% glutaraldehyde stationary liquid at 4 °C for 4 h were placed on low speed centrifugation, wrapped in 1% agar-sugar, and washed in PBS. The tissues were post-fixated in a 1% osmium tetroxide solution for 2 h at 20 °C, and dehydrated in an ascending series of ethanol and acetone containing solutions. Tissues were then embedded in Epon812 resin. The ultrathin sections (60–80 nm thick) were made and contrasted with a 1% mixture of uranyl acetate combined with lead citrate. These units were then observed using a TEM (HITACHI HT7700, Japan).

### Immunohistochemistry

After dewaxing, the sections were heated in citrate buffer (PH 6.0) for 15 min, chilled, and washed with PBS. Units were maintained at room temperature in a 3% hydrogen peroxide mixture to block endogenous peroxidase activity. Then, sections were maintained overnight in several different principal antibodies: rabbit anti-VDR antibodies (VDR antibody, 14,526–1-AP, Proteintech, USA, diluted at 1:200); rabbit anti-CYP27B1 antibodies (anti-CYP27B1, ab206655, Abcam, UK, diluted at 1:2000); rabbit anti-CYP19 antibodies (cytochrome P450 19A1 antibody, DF3564, Affinity, diluted at 1:50–200); rabbit anti- HOXA10 antibodies (HOXA10 rabbit polyclonal antibody, 26,497–1-AP, Proteintech, USA, diluted at 1:200). After rinsing with PBS, appropriate secondary antibodies were applied for 50 min to perform the detection. The sections were rinsed with PBS, used for DAB color rendering, and the nuclei were restained with hematoxylin for approximately 3 min. The slides were then dehydrated, airdried, and coverslipped. Image-Pro Plus 6.0 software was used to quantify and analyze the positive signals. Each photo was analyzed to obtain the cumulative integrated optical density (IOD) of positive signals and tissue’s pixel area (AREA). Finally, the area density was calculated by IOD / AREA. A larger areal density value indicates a higher positive expression.

The control slides were created using sections of the endometrial tissue. These slides were treated identically to the others, except that the primary antibodies were replaced with the corresponding primary isotype rabbit IgG antibodies to control for non-specific staining.

### Western blot analysis

A Minute™ Cytoplasmic and Nuclear Fractionation kit (SC-003, Invent, USA) was used for tissue protein extraction. Aliquots were assayed for protein content using the BCA method. Samples were separated by 12% SDS-PAGE under reducing and electroblotted onto a PVDF membrane. They were then blocked in TBS+ 0.1% TBST containing 5% non-fat powdered milk and shaken for 60 m. Membranes were incubated with VDR (14526–1-AP, Proteintech, USA), CYP27B1 (ab206655, Abcam, UK), CYP19 (DF3564, Affinity, China), HOXA10 (26497–1-AP, Proteintech, USA), GAPDH (ab9485, Abcam, UK) and H3 (ab1791, Abcam, UK) primary antibodies at 4 °C overnight. The resulting antibody solution was a 1:1000 dilution. Membranes were then bathed for 5 min in TBST three separate times, then incubated with horseradish peroxidase (HRP), a conjugated secondary antibody (Servicebio, China), at a dilution of 1:3000, for 30 min. Membranes were then washed. Enhanced chemiluminescence was used for exposure imaging. Photos of the bands were captured and the density of each band was determined using Image Alpha software. The ratio of each cytoplasmic protein /GAPDH and nuclear protein/Histone H3 band density was calculated and considered as an indicator for expression level in the targets.

### Statistical analysis

We completed all analyses using the SPSS statistical software package (13.0 version, Chicago, IL). We determined variable normality utilizing the Kolmogorov–Smirnov test. We displayed continuous variables using the associated median and quantiles or mean ± the standard error. Normally distributed variables were analyzed using one-way ANOVA. The Brown-Forsythe test was used when the distribution of variables did not meet the requirement of homogeneity of variance. For irregularly dispersed variables, we used the Mann–Whitney test for intragroup comparisons. The correlation between continuous variables was determined with Spearman correlation analysis. Bonferroni’s correction was used for multiple testing. A *p*-value of 0.05 was used as the threshold for statistical significance.

## Results

### Patient characteristics

We recruited a total of 16 participants for our analysis. The mean age of participants was 34.6 years old. There were 8 cases each in the proliferative and secretory endometrial phases. Other factors that were comparable between patients in the proliferative and secretory groups include sub-category of infertility (primary or secondary), cause of infertility (male factor or tubal factor), infertility duration, and quantity and quality of embryos moved in the initial frozen-thawed embryo transfer sequence.

Patients’ baseline characteristics and IVF outcomes of the two groups are summarized in Table 1. No substantial variance in mean age (*P* = 0.685), BMI (*P* = 0.294), serum 25(OH) D levels (*P* = 0.67), thickness of endometrium on the day of embryo transfer (*P* = 0.656), or clinical pregnancy rate (*P* = 0.626) was detected between the two study groups.

**Table 1:** Baseline and IVF variables grouped by endometrial phase (median and quantiles)

Variable	Proliferative phase (*n* = 8)	Secretory phase (n = 8)	*p*-value
Age (years)	35.5 (33.5–36.0)	35.0 (29.0–40.0)	0.685
BMI (kg/m^2^)	19.95 (18.9–21.5)	21.1 (19.4–22.8)	0.294
Serum 25(OH) D (ng/ml)	13.5 (9.56–17.6)	14.7 (8.52–16.2)	0.670
Thickness of endometrium on day of embryo transfer (mm)	11.8 (9.63–12.0)	12.0 (10.0–13.0)	0.656
Clinical pregnancy n (%)	4 (50)	3 (37.5)	0.626

*BMI*: Body mass index.

### SEM and TEM microphotographs of endometrium under different concentrations of vitamin D influence during the window of implantation

**Fig. 1:**
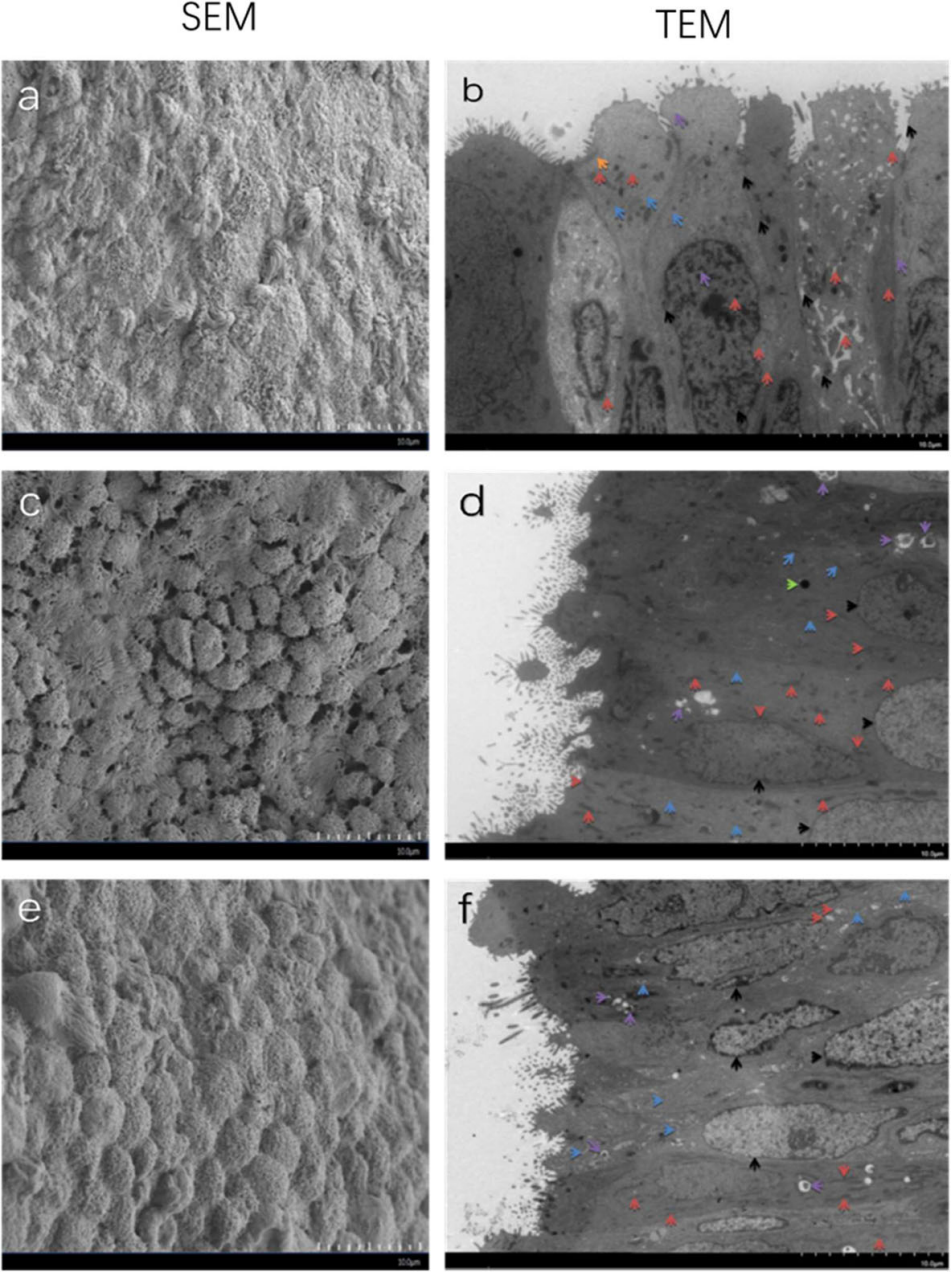
SEM and TEM microphotographs of the endometrium under the influence of different concentrations of vitamin D during the implantation window. SEM and TEM microphotographs of vitamin D deficient (**a** and **b**), insufficient (**c** and **d**), and replete (**e** and **f**) patients. (**a**) Pinopodes not yet fully formed, ×3000 magnification. (**c**) Pinopodes in the developmental stage with abundant expression, × 3000 magnification. (**e**) Clusters of mature pinopodes with abundant expression, × 3000 magnification. **b** Intracytoplasmic organelles abundant with a few vacuoles; The nucleus is irregularly shaped and locally concave. Heterochromatin increases and aggregates can be seen around the nuclear envelope (examples are indicated by black arrowheads); The mitochondria are abundant, some are dropsical and dilated (reddish brown); The rough endoplasmic reticulum is swollen and dilated (blue); The Golgi apparatus (orange); Intracellular autophagy are less distributed (purple). **d** Intracytoplasmic organelles are abundant with a few vacuoles; The nucleus is irregularly shaped and locally concave without heterochromatin (black); The mitochondria are abundant without obvious edema or distention (reddish brown); The rough endoplasmic reticulum (blue); Intracellular autophagy is obviously increased (purple). The lysosomal (green). **f** Intracytoplasmic organelles are abundant; The nucleus is irregularly shaped and some are accompanied by nuclear division. Heterochromatin levels are elevated and aggregated around the nuclear envelope (black); The mitochondria are abundant, some are dropsical and dilated (reddish); The rough endoplasmic reticulum is swollen and dilated (blue); Intracellular autophagy is obviously increased (purple)

A SEM was used to observe the expression and morphology of the pinopodes in women with vitamin D deficiency, insufficiency, and repletion. Additionally, a TEM was used to further confirm the ultrastructure (Fig. 1). Pinopodes occur specifically in the endometrium during the window of implantation. Morphologically, the appearance of mature pinopodes is the best indicator of endometrial receptivity. The results of SEM analysis indicated that higher vitamin D levels were associated with more mature pinopodes. For vitamin D deficient patients, the surface of the luminal epithelium was flat, and microvilli were seen on the cell surface. In addition, pinopodes were not yet fully formed and only slightly expressed in vitamin deficient subjects. For vitamin D insufficient patients, the surface of the luminal epithelial cells were raised, displayed abundant microvilli, and the pinopodes during the developmental phase were richly expressed. For vitamin D replete patients, the epithelial cell surfaces were bulbous, smooth, and the microvilli had been shed. There were clusters of mature pinopodes undergoing abundant expression in the vitamin D replete subjects. The results of TEM showed that mitosis and autophagy increased as vitamin D concentrations increased. The distribution and morphology of the mitochondria and rough endoplasmic reticulum were not significantly different.

### Target protein distribution in different phases of endometrium

**Fig. 2:**
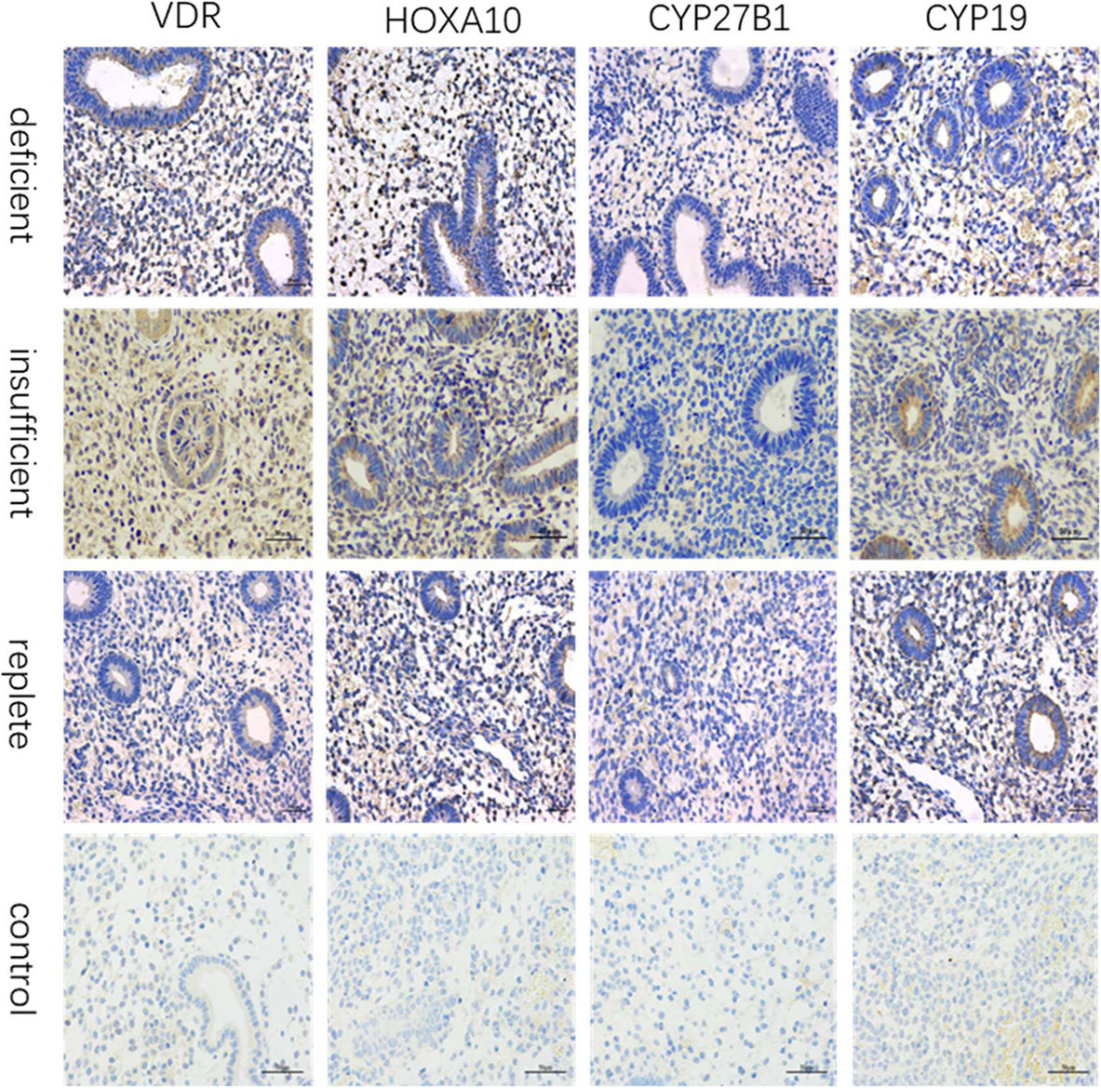
Immunohistochemistry localization of VDR (left column), HOXA10 (second column), CYP27B1 (third column), and CYP19 (right column) during proliferative phase of the menstrual cycle in vitamin D deficient (top row), insufficient (second row), and replete (third row) patients. No signal was detected in the negative control sections (bottom row). All parts: magnification × 400, scale bar 50 μm

**Fig. 3:**
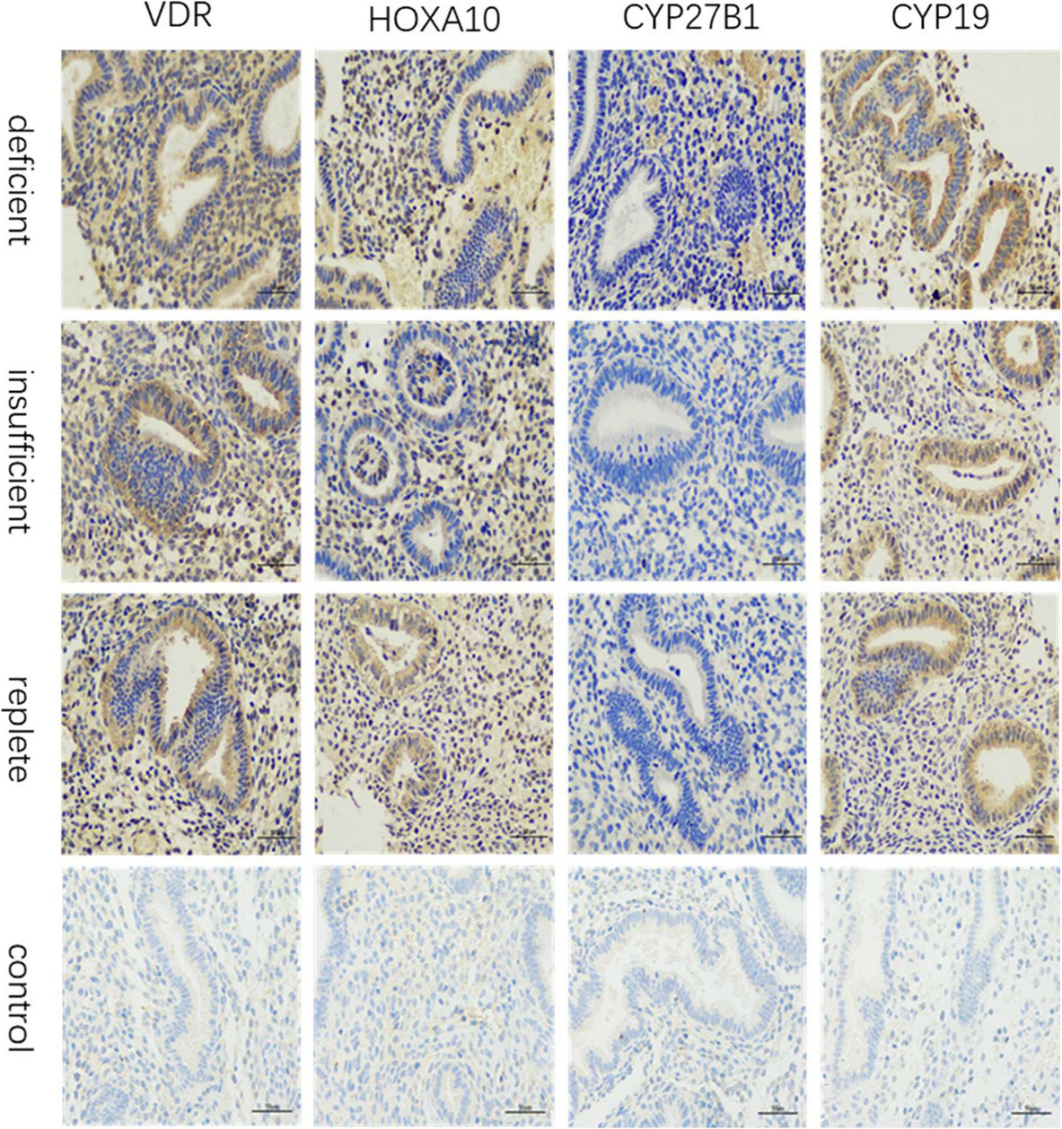
Immunohistochemistry localization of VDR (left column), HOXA10 (second column), CYP27B1 third column), and CYP19 (right column) during the secretory phase of the menstrual cycle in vitamin D deficient (top row), insufficient (second row), and replete (third row) patients. No signal was detected in the negative control sections (bottom row). All parts: magnification × 400, scale bar 50 μm

We performed immunohistochemistry to determine the localized effects of VDR, HOXA10, CYP27B1, and CYP19 protein expression in the endometrium from the proliferative phase (Fig. 2) to the secretory phase (Fig. 3). As shown, Figs. 2 and 3 are representative photos. Overall, VDR (Figs. 2 and 3, left column), HOXA10 (Figs. 2 and 3, second column), CYP27B1 (Figs. 2 and 3, third column) and CYP19 (Figs. 2 and 3, right column) expression were detected in all endometrial tissue samples. The VDR protein was observed intracellularly and at the plasma membrane level. It was predominantly localized to the nucleus of the endometrial cells and was detected in the uterine glandular epithelium and stromal cells during menstruation. Area density analysis for immunohistochemical positive signals suggested, a relatively higher VDR distribution was observed in the endometrial tissue of women with high levels of serum vitamin D (results not shown). The HOXA10 protein was predominantly concentrated in the nucleus of the endometrial cells and was detected in the uterine glandular epithelium and stromal cells during the menstrual cycle. A relatively higher HOXA10 distribution was observed during the secretory phase. The immunostain of CYP27B1 and CYP19 enzymes was mainly localized in the cytoplasm of epithelium and stromal cells.

### Target protein expression in different phases of endometrium

**Fig. 4:**
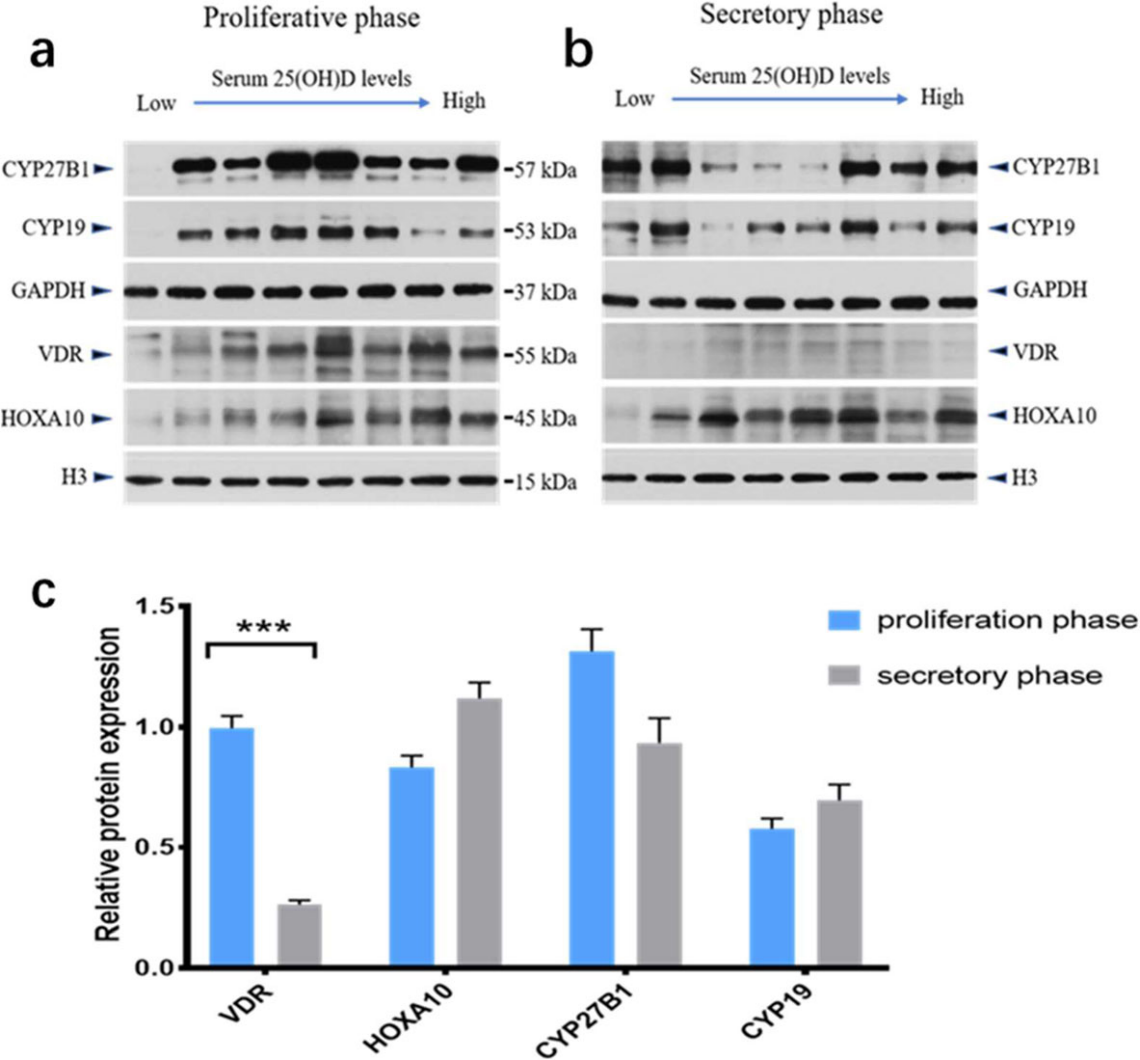
Western blot for VDR, CYP27B1, HOXA10, and CYP19 during the proliferative (**a**) and secretory (**b**) phases of the menstrual cycle. Summarized data are presented as the mean ± SEM of eight observations in each group, ***P < 0.001 (c)

We investigated the tissue expression of VDR, CYP27B1, HOXA10 and CYP19 during different phases of the human menstrual cycle using western blot (Fig. 4). In general, all four target proteins were expressed in the endometrium during different phases. Expression of VDR proteins during the endometrial secretory phase was significantly reduced contrasted with those in the proliferation phase (*P <* 0.001). Compared with the proliferative phase, HOXA10 protein manifestation increased in the secretory phase, but the difference was not statistically significant (*P* = 0.23). No substantial variance in protein expression of CYP27B1 and CYP19 were observed when comparing the proliferative and secretory phases (*P* = 0.352 and *P* = 0.588, respectively). Area density analysis for immunohistochemical positive signals also suggested the similar results (Additional file 1: Supplementary figure).

### Target protein expression according to clinical pregnancy status

**Fig. 5:**
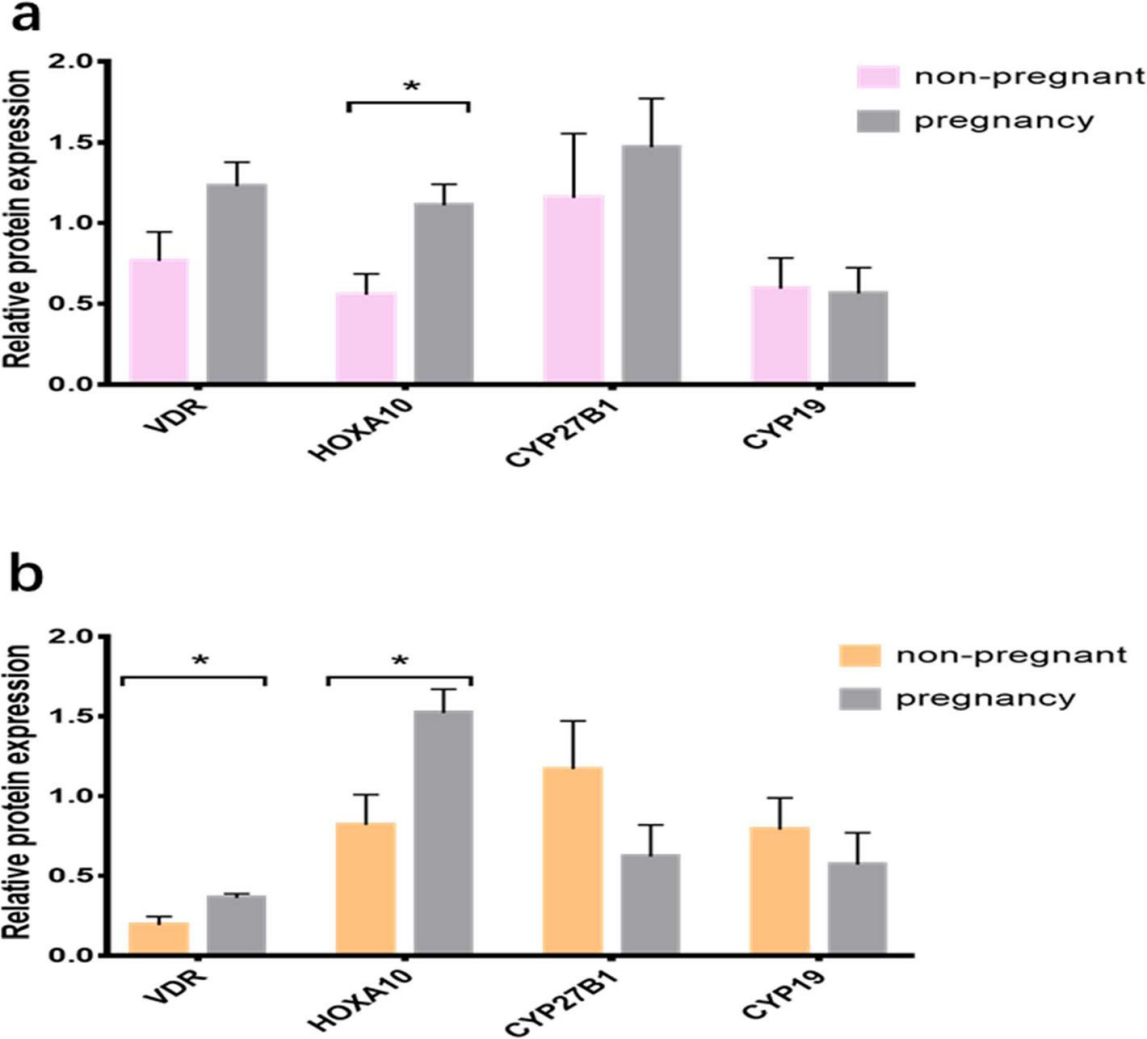
Target protein expression according to clinical pregnancy status. Comparison of target protein expression between pregnant and non-pregnant patients during the proliferative phase (**a**) and during the secretory phase (**b**) of the menstrual cycle. Summarized data are presented as the mean ± SEM of three to four observations in each subgroup, *P < 0.05

Next, we grouped patients according to pregnancy outcomes, compared the differences in the expression of target proteins by outcome, and investigated the correlation between each target protein. Figure 5a compares the target protein expression between pregnant and non-pregnant patients during the proliferative phase. Serum 25(OH) D levels (*P* = 0.149) and mean patient age (*P* = 0.102) were comparable between the two subgroups. HOXA10 protein expression in pregnant women increased significantly when measured against non-pregnant women (*P* < 0.023). Compared with non-pregnant women, VDR protein expression increased in pregnant women, but the disparity was not statistically significant (*P* = 0.083). We did not find a substantial discrepancy in the protein expression of CYP27B1 and CYP19 between the two subgroups (*P* = 0.773 and *P* = 0.885, respectively). Moreover, the spearman correlation suggests that VDR protein expression was significantly positively correlated with HOXA10 protein expression (*R* = 0.868, *P* = 0.005), serum 25(OH) D levels were meaningfully positively correlated with HOXA10 protein expression (*R* = 0.768, *P* = 0.026), and CYP21B1 protein expression was significantly correlated with increased CYP19 protein expression (*R* = 0.76, *P* = 0.029).

Figure 5b shows the comparison of the relevant protein expression between pregnant and non-pregnant patients during the secretory phase of the menstrual cycle. Serum 25(OH) D levels (*P* = 0.289) were comparable between the two subgroups, while pregnant women were significantly younger than their non-pregnant counterparts (*P* = 0.032). As Table 2 shows, both HOXA10 and VDR protein expression in pregnant women were significantly elevated compared to their non-pregnant counterparts (*P* = 0.034 and *P* = 0.038). No meaningful variation in the protein expression of CYP27B1 and CYP19 existed between the two subgroups (*P* = 0.289 and *P* = 0.593). Moreover, once we adjusted for age, correlation analysis showed that VDR protein expression was significantly positively correlated with HOXA10 protein expression (*R* = 0.807, *P* = 0.005), serum 25(OH) D levels were meaningfully positively correlated with VDR protein expression (*R* = 0.744, *P* = 0.09), and CYP21B1 protein expression was significantly associated with CYP19 protein expression (*R* = 0.816, *P* = 0.047).

**Table 2:** Correlation analysis between pairs of target molecules during proliferative and secretory phases of endometrium

Pair	proliferative phase		secretory phase			
	*r*	*p*-value	*r*	*p*-value	*r*^*1*^	*p*^*1*^-value
VDR vs HOXA10	0.868	0.005	0.946	0.001	0.807	0.05
VDR vs CYP27B1	0.494	0.213	−0.764	0.046	−0.836	0.038
VDR vs CYP19	0.285	0.494	−0.266	0.564	−0.471	0.346
VDR vs 25(OH)D	0.529	0.177	0.491	0.263	0.744	0.09
HOXA10 vs CYP27B1	0.613	0.106	−0.571	0.18	−0.41	0.42
HOXA10 vs CYP19	0.269	0.52	−0.18	0.699	0.043	0.935
HOXA10 vs 25(OH)D	0.768	0.026	0.607	0.148	0.703	0.119
CYP27B1 vs CYP19	0.76	0.029	0.667	0.102	0.816	0.047
CYP27B1 vs 25(OH)D	0.403	0.322	−0.321	0.482	−0.556	0.252
CYP19 vs 25(OH)D	0.074	0.861	−0.487	0.268	−0.489	0.325

^*1*^*r* value and *p* value after adjusting for age that was associated with VDR and HOXA10 during secretory phase of endometrium

## Discussion

This study is unique in that it concomitantly measured the change in expression of endometrial VDRs and vitamin D metabolizing enzymes during the menstrual cycle of women of reproductive age. In addition, this study explored the relationship between the expression of the vitamin D-VDR system and endometrial receptivity in the implantation window. All molecules of interest were expressed in endometrial samples. VDR and HOXA10 expression significantly increased during the secretory phase in pregnant women contrasted with non-pregnant women. Our findings suggest that the vitamin D-VDR system is extensively linked to the regulation of endometrial receptivity thereby further affecting pregnancy outcomes.

VDR expression has been demonstrated at different phases of the human menstrual cycle. However, the difference in expression between the proliferative and secretory phases remains controversial. Vienonen et al. first reported no significant difference in VDR gene expression between different phases of the menstrual cycle in premenopausal women [20]. Bergadà et al. reported that VDR was upregulated while during the secretory phase in comparison to the proliferative phase in an unselected population [21]. Zelenko et al. studied VDR expression in women with and without endometriosis. Interestingly, they found VDR downregulation while in the mid secretory phase as opposed to the early secretory phase, but no notable difference was found compared with the proliferative phase in endometrium absence of endometriosis. However, VDR expression did not differ from proliferative phase to secretory phase in endometrium in the presence of endometriosis [22]. Our research focused on a specific fertility group, young women undergoing embryo transfer. Similar to Zelenko’s study, we found that the protein expression of VDR in the secretory phase was significantly reduced compared to the proliferative phase in these women.

The contradictory outcomes described in prior research might suggest that exposure of the endometrium to different disease conditions or different study populations appears to influence vitamin D signaling pathways. VDR is part of a larger group of transcriptional regulators known as nuclear receptors, and facilitates the diverse biological impacts of 1,25(OH)_2_D and related compounds [23]. While we know that VDR controls thousands of genes, much remains unclear about the specific mechanisms underlying the management of the VDR genetic factor itself. VDR regulation under basal conditions and upon induction is multi-layered; shaped by the environment; and influenced by genetics and epigenetics [24]. Future research needs to explore the mechanism underling changes of VDR expression during the menstrual cycle. Additionally, it should be noted that our study has compared different women with different menstrual cycle phases, thereby raising the question of how many of the described changes are due to true biological changes and not inter-individual variability. This is needed to be confirmed in future.

Interestingly, we found that higher serum vitamin D levels were associated with more mature pinopodes during the implantation window. VDR and HOXA10 (endometrial receptivity marker) protein expression were higher in pregnant women compared to non-pregnant women, and VDR protein levels were positively correlated with HOXA10 levels. In addition, serum vitamin D levels were positively correlated with VDR and HOXA10 protein levels in endometrium. These results suggest that high expression of the vitamin D-VDR complex in the endometrium, especially during the implantation window, is more conducive to the formation of endometrial receptivity and the establishment of pregnancy.

Research suggests that Vitamin D has beneficial effects on the process of implantation. Rudick and colleagues first reported that individual vitamin D status was related to IVF success in donor-recipient cycles, which suggested that the endometrium might mediate the impacts of vitamin D, since the oocyte donor-recipient model was able to distinguish the influence of vitamin D on oocytes vs. the endometrium [25]. Biological evidence underlying this process is still limited. Vitamin D may induce differentiation during the secretory phase of the menstrual cycle through activation of classic developmental modulators, such as HOX genes. Du Hongling’s research, using primary human endometrial stromal cells, found that 1,25(OH)_2_D_3_ prompts HOXA10 transcription through the binding of VDR to a VDRE in the HOXA10 gene 5′ region [7]. In addition, Evans established that many elements of vitamin D signaling and metabolic breakdown are robustly expressed in human uterine decidua, signifying that 1,25(OH)_2_D_3_ production in the mucous membrane may exert immunosuppressive effects during the start of gestation [26].

Previous studies have confirmed that endometrial expression of the mitochondrial enzyme 1-hydroxylase is encoded by CYP27B1 [4]. However, quantification of this expression throughout menstruation has not yet been performed. In our study, we found no significant variation in the expression of CYP27B1 in women at different phases of menstruation or with different pregnancy outcomes, and there was no detectable link related to the expression of VDR or HOXA10. Many key enzymes are involved in the vitamin D metabolic pathway, during synthesis and inactivation for example. One of the limitations of this study is that we only tested one enzyme, CYP27B1. Subsequent studies should continue to concentrate on the metabolic changes of the vitamin D pathway in the endometrium. In addition, our data suggests that the expression of CYP27B1 is closely related to CYP19. We speculate that metabolic pathways associated with vitamin D are involved in the regulation of steroidogenesis in the endometrium, which further affects endometrial function.

Our results suggest that the vitamin D-VDR system is very important with regard to receptivity of human endometrial tissue. The biggest limitation of this study was the limited sample size. Therefore, true differences between the groups due to underlying medical conditions (rather than the endometrial phase) cannot be excluded. Additionally, there are several caveats to our findings. Although the collected endometrium samples were acquired during different phases of the menstrual cycle, we were not able to collect samples from the same patient during each phase of the menstrual cycle due to the precious of the specimens and invasiveness of the operation. Although the histological research shows a significant correlation between VDR and HOXA10 expression, the lack of functional data from an in-vitro model is a limitation of this study. Thus, more in vivo research into the signaling pathways involved in the VDR-induced up-regulation of HOXA10 is sorely needed.

## Conclusion

We demonstrated that several vitamin D metabolic and signaling molecules are generated by human endometrial, epithelial, and stromal cells, and that VDR expression is cycle-dependent, implying a key role for the Vitamin D-VDR system within the endometrium. Additionally, increased expression of VDR in the endometrium, especially during the implantation window, was more conducive to the formation of endometrial receptivity and the establishment of pregnancy. Therefore, clinical studies are required to evaluate the effect of vitamin D supplement on patients with impair endometrial receptivity, like repeated implantation failure or repeated abortion. This may help to develop a potential vitamin D therapeutic application to improve reproductive outcomes in women with infertility.

## Supplementary information

**Additional file 1: Supplementary figure.** Area analysis method for immunohistochemistry for for VDR, CYP27B1, HOXA10, and CYP19 during the proliferative and secretory phases of the menstrual cycle.

## Data Availability

The datasets analyzed during the current study are available from the corresponding author on reasonable request.

## Abbreviations

1,25(OH)_2_D: 1,25-dihydroxyvitamin D
25(OH)D: 25-hydroxyvitamin D
AREA: the tissue’s pixel area
BMI: Body mass index
COS: Controlled ovarian stimulation
E_2_: Estradiol
HCG: Human chorionic gonadotrophin
IOD: Integrated optical density
IOM: Endocrine Society and Institute of Medicine
IVF-ET: In vitro fertilization and embryo-transfer
LH: Luteinizing hormone
PBS: Phosphate buffer saline
SD: Standard deviation
SEM: Scanning electron microscope
SEM: Standard error of the mean
SPSS: Statistical package for the social sciences
TEM: Transmission electron microscope
VDR: Vitamin D receptor
VDRE: Vitamin D response element
