# Supplementary material for: Characterization of VDR and CYP27B1 expression in the endometrium during the menstrual cycle before embryo transfer: implications for endometrial receptivity
Source: Reprod Biol Endocrinol. 2020 Mar 17;18:24. doi: 10.1186/s12958-020-00579-y (PMC7079352; doi:10.1186/s12958-020-00579-y)
Supplement: Supplementary file 1 — Additional file 1: Supplementary figure. Area analysis method for immunohistochemistry for for VDR, CYP27B1, HOXA10, and CYP19 during the proliferative and secretory phases of the menstrual cycle. [file 12958_2020_579_MOESM1_ESM.docx]

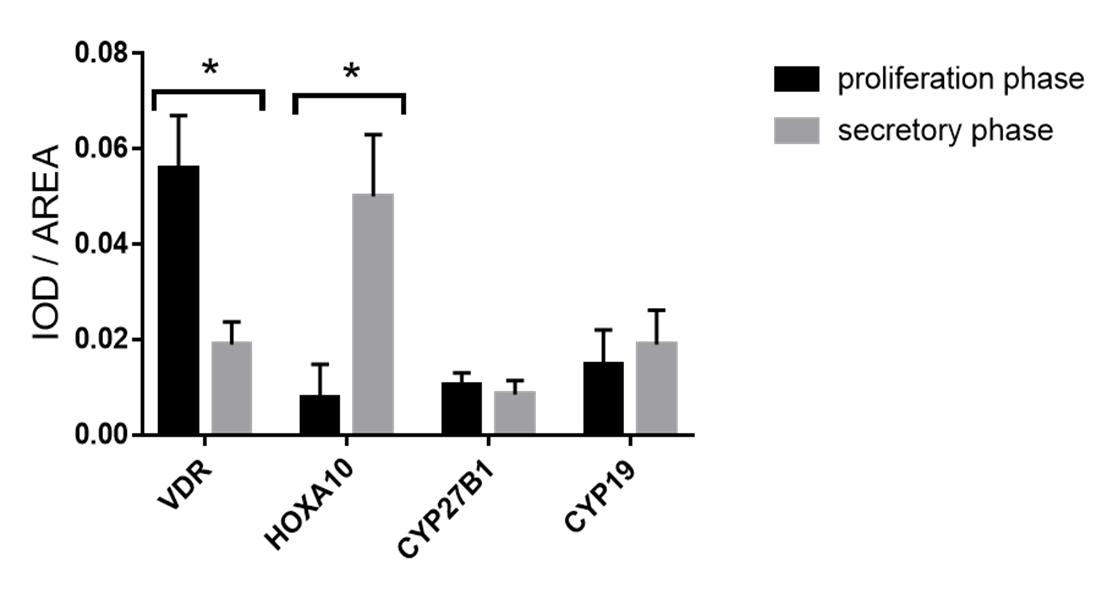


Supplementary figure

Area analysis method for immunohistochemistry for for VDR, CYP27B1, HOXA10, and CYP19 during the proliferative and secretory phases of the menstrual cycle. Summarized data are presented as the mean ± SEM of eight observations in each group, *P < 0.05.
